# Robotic thoracic surgery for inflammatory and infectious lung disease: initial experience in Brazil

**DOI:** 10.1590/0100-6991e-20202872

**Published:** 2021-05-05

**Authors:** PEDRO HENRIQUE CUNHA LEITE, ALESSANDRO WASUM MARIANI, PEDRO HENRIQUE XAVIER NABUCO DE ARAUJO, CARLOS EDUARDO TEIXEIRA LIMA, FELIPE BRAGA, RUI HADDAD, JOSÉ RIBAS MILANEZ DE CAMPOS, PAULO MANUEL PEGO-FERNANDES, RICARDO MINGARINI TERRA

**Affiliations:** 1 - Hospital São Rafael, Serviço de Cirurgia Torácica - Salvador - BA - Brasil; 2 - Instituto do Coração, Faculdade de Medicina, Universidade de São Paulo, Departamento de Cirurgia Torácica - São Paulo - SP - Brasil; 3 - Rede D’Or - São Luiz, Serviço de Cirurgia Torácica - São Paulo - SP - Brasil; 4 - Hospital Sírio Libanês, Serviço de Cirurgia Torácica - São Paulo - SP - Brasil; 5 - Hospital Copa Star, Serviço de Cirurgia Torácica - Rio de Janeiro - RJ - Brasil

**Keywords:** Infectious Lung Disease, Bronchiectasis, Robotic Surgery, Thoracic surgery, Doença Pulmonar Infecciosa, Bronquiectasia, Cirurgia Robótica, Cirurgia Torácica

## Abstract

**Objective:**

in Latin America, especially Brazil, the use of a robotic platform for thoracic surgery is gradually increasing in recent years. However, despite tuberculosis and inflammatory pulmonary diseases are endemic in our country, there is a lack of studies describing the results of robotic surgical treatment of bronchiectasis. This study aims to evaluate the surgical outcomes of robotic surgery for inflammatory and infective diseases by determining the extent of resection, postoperative complications, operative time, and length of hospital stay.

**Methods:**

retrospective study from a database involving patients diagnosed with bronchiectasis and undergoing robotic thoracic surgery at three hospitals in Brazil between January of 2017 and January of 2020.

**Results:**

a total of 7 patients were included. The mean age was 47 + 18.3 years (range, 18-70 years). Most patients had non-cystic fibrosis bronchiectasis (n=5), followed by tuberculosis bronchiectasis (n=1) and lung abscess (n=1). The performed surgeries were lobectomy (n=3), anatomic segmentectomy (n=3), and bilobectomy (n=1). The median console time was 147 minutes (range 61-288 min.) and there was no need for conversion to open thoracotomy. There were no major complications. Postoperative complications occurred in one patient and it was a case of constipation with the need for an intestinal lavage. The median for chest tube time and hospital stay, in days, was 1 (range, 1-6 days) and 5 (range, 2-14 days) respectively.

**Conclusions:**

robotic thoracic surgery for inflammatory and infective diseases is a feasible and safe procedure, with a low risk of complications and morbidity.

## RESUMO

**Objetivo:**

na América Latina, especialmente no Brasil, a adoção da plataforma robótica para cirurgia torácica está aumentando gradativamente nos últimos anos. No entanto, apesar da tuberculose e doenças pulmonares inflamatórias serem endêmicas em nosso país, faltam estudos que descrevam os resultados do tratamento cirúrgico robótico das bronquiectasias. Este estudo tem como objetivo avaliar os resultados cirúrgicos da cirurgia robótica para doenças inflamatórias e infecciosas, determinando a extensão da ressecção, complicações pós-operatórias, tempo operatório e tempo de internação hospitalar.

**Métodos:**

estudo retrospectivo a partir de um banco de dados envolvendo pacientes com diagnóstico de bronquiectasia e submetidos à cirurgia torácica robótica em três hospitais brasileiros entre janeiro de 2017 e janeiro de 2020.

**Resultados:**

foram incluídos 7 pacientes. A média de idade foi 47 + 18,3 anos (variação, 18-70 anos). A maioria dos pacientes apresentou bronquiectasia não fibrose cística (n=5), seguida de bronquiectasia tuberculosa (n=1) e abscesso pulmonar (n=1). As cirurgias realizadas foram lobectomia (n=3), segmentectomia anatômica (n=3) e bilobectomia (n=1). O tempo médio do console foi de 147 minutos (variação de 61-288 min.) e não houve necessidade de conversão para toracotomia. Complicação pós-operatória ocorreu em um paciente, tratando-se de obstipação com necessidade de lavagem intestinal. A mediana do tempo de drenagem torácica e internação hospitalar, em dias, foi de 1 (variação, 1-6 dias) e 5 (variação, 2-14 dias), respectivamente.

**Conclusões:**

a cirurgia torácica robótica para doenças inflamatórias e infecciosas é um procedimento viável e seguro, com baixo risco de complicações e morbidade.

## INTRODUCTION

Despite the advancement and spread of videothoracoscopy (VATS) from the 90’s on^1^, the surgical treatment of lung inflammatory and infectious diseases is still strongly associated with classic thoracotomy. Diseases such as tuberculosis, aspergilloma, and bronchiectasis destroy the lung parenchyma, promoting formation of intense pleuropulmonary adhesions, pulmonary hilum freezing, and calcification of the mediastinal lymph nodes^2^, making the adoption of minimally invasive methods a challenge to the surgeon and limiting the use in such cases^2^ ^,^ ^3^.

Despite the technical difficulties, such as the 2D view and non-articulated instruments^4^, VATS is used in some centers in the surgical treatment of infectious lung diseases, with low perioperative morbidity associated with the advantages of the minimally invasive technique^5^ ^,^ ^6^.

 More recently, robotic surgery has emerged as an alternative to VATS, providing the surgeon with 3D vision and wider and more precise movements within the thoracic cavity^7^. Given these technical advantages, we believe that the robotics platform would be of paramount importance to the aid and the expansion of minimally invasive methods in surgical treatment of lung inflammatory and/or infectious conditions. The first robotic-assisted lobectomy for this indication was performed in 2013 in Saint Petersburg, Russia, by Yablonskiii et al.^8^. Since then, few studies have been published on the technical aspects and perioperative outcomes of lung resection in bronchiectasis^2^ ^,^ ^9^.

In Brazil, the use of the robotics platform for thoracic surgery is already a reality, being common in various centers. However, even though tuberculosis and other infectious and inflammatory lung diseases are endemic in the country, there are no national studies describing the results of robotic-assisted surgical treatment for such diseases. The aim of this study is to address the technical aspects of the procedure and to evaluate the postoperative result of robotic surgery in the treatment of lung infectious and inflammatory diseases by analyzing the extent of resection, postoperative complications, surgical time, and hospital stay.

## METHODS

This is a retrospective study of patients with pulmonary inflammatory disease, associated or not with infectious bronchiectasis, undergoing lung resection with the aid of a robotic platform.

This study was approved by the Ethics in Research Committee of the institution, with reference number NP 1445/18.

All procedures were performed by the same group of surgeons in three hospitals in Brazil: Hospital São Luiz Itaim (SP), Hospital Sirio-Libanes (SP), and Hospital Copa Star (RJ). The robotic platform used was the da Vinci ® Intuitive Surgical System (Intuitive Surgical Inc., Sunnyvale, CA, USA).

The operations were conducted in the period between February 2017 and September 2019. The data were collected from a unique robotic surgery database and updated regularly by our team. We included all patients undergoing lung resection for inflammatory disease, associated or not with infectious bronchiectasis, since the start of the group, in 2015.

 The eligibility criteria for pulmonary resection were patients with localized disease who, despite clinical treatment, maintained signs and symptoms, such as recurrent pulmonary infection and/or hemoptysis.

**Figure 1:**
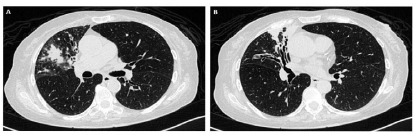
Female patient, 70 years old, diagnosed with bronchiectasis associated with Mycobacterium abscessus. In A and B there is a cut axial CT scan of the chest with thickening of the bronchial walls, especially in the right upper and middle lobe associated with foci of consolidation and irregular focal opacities.

The preoperative evaluation consisted of chest-computed tomography (CT), bronchoscopy with collection of bronchoalveolar lavage, cardiac evaluation, complete pulmonary function test, and laboratory tests. Figure 1 shows a patient’s tomography with bronchiectasis.

The surgical procedure was standardized in all cases. All operations were carried out with selective intubation confirmed by bronchoscopy. We did not use routine epidural catheter, analgesia taking place in a preemptive manner, ie, local anesthesia before the first incision and after the introduction of the camera, and blockade of the 4th to the 11th intercostal space under direct vision.

**Figure 2:**
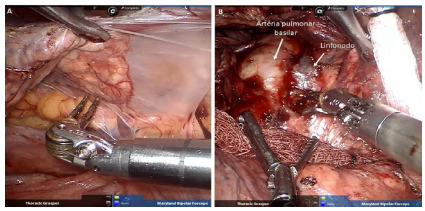
Intraoperative image of right upper bilobectomy to bronchiectasis secondary to Mycobacterium abscessus. In A, we observed the presence of pleuropulmonary adhesions with the chest wall and mediastinum. In B, the presence of an inflammatory lymph node attached to the basilar pulmonary artery is noted.

The operation is performed systematically to minimize pulmonary manipulation, as already described by Terra et al.^9^. As a technical detail, is cases of pleuropulmonary adhesions found during inspection of the pleural cavity after introduction of the optics, before docking of the robotic platform we chose to release the lung by thoracoscopy until the trocars’ puncture sites for the robotic arms were free of adhesions. Figure 2 shows an intraoperative image of lung resection due to bronchiectasis.

 In this study, we evaluated variables related to the surgical technique (length of surgical procedure and extension of resection) and perioperative outcomes (length of ICU stay, hospital stay, and pleural drainage, in addition to the presence of postoperative complications).

We measured the total operative time by the time interval between the skin incision until closure, including the following surgical times: incision and establishment of surgical portals; robot docking; console time; and chest wall closure time. We measured the time lengths of drain, ICU, and hospital stay in days (D) from the day of the operation, this being called D0, and the subsequent days, D1, D2 and so on. We considered the day of hospital discharge the day on which the patient left the hospital, regardless of the time of discharge.

### Statistical analysis

 We represented continuous variables as mean and standard deviation or as median and interquartile range (IQR). We expressed categorical variables in absolute numbers and proportions. We set the Type I error at 5%.

## RESULTS

During the study period, we included seven patients, five of them women, with an average age of 47.0 ± 18.3 years (range 18 to 70). Only two patients had comorbidities, one case of ischemic heart disease and another with a history of breast cancer. Most patients had bronchiectasis without cystic fibrosis (n = 5), followed by one case of tuberculosis sequelae and one of bronchiectasis associated with Mycobacterium abscessus.

All procedures were anatomical pulmonary resections, with three pulmonary lobectomies, three segmentectomies, and one bilobectomy. The average total surgical time was 212 minutes, varying from 100 to 375, and the average console time was 147 minutes, ranging from 61 to 288. There was no conversion to thoracotomy or intraoperative complication. Perioperative bleeding was estimated to be less than 100 mL for all cases. This value was obtained from measuring the volume in the suction container and counting the gauze pods; however, as the gauzes were not weighed, only counted, this statement is subjective.

Only two patients were referred to the intensive care unit after the operation, one of them due to advanced age and the presence of comorbidities, and the other, according to the hospital’s protocol. The median length of stay in the ICU was zero days, ranging from zero to five. The median thoracic drainage time was one day, ranging from one to six, since the median hospital stay was five days, varying between two and 14 days. There were no major complications. The only postoperative complication was one case of constipation that required an intestinal lavage. Table 1 comprises the summary of patients’ clinical profiles and postoperative outcomes.

**Table 1:** Clinical profiles and postoperative outcomes..

Case	Age (years)	Gender	Diagnosis	Procedure	ICU (days)	Drainage (days)	In-hospital (days)	Complications
Case 1	43	F	Tb sequelae	Lobectomy	4	3	3	None
Case 2	57	F	Bronchiectasis	Segmentectomy	0	1	2	None
Case 3	70	F	NTM*	Bilobectomy	5	4	14	None
Case 4	32	M	Bronchiectasis	Lobectomy	0	6	7	None
Case 5	36	M	Bronchiectasis	Lobectomy	0	1	2	None
Case 6	18	F	Bronchiectasis	Segmentectomy	0	1	5	None
Case 7	70	F	Bronchiectasis	Segmentectomy	0	1	6	Yes#

* NTM - Non-tuberculous mycobacteria.

# Constipation

## DISCUSSION

Historically the classical approach to the surgical treatment of inflammatory lung disease is thoracotomy, and thoracic surgeons agree that this operation is technically difficult and challenging, especially due to the presence of pleuropulmonary adhesions.

Pulmonary detachment and lysis of adhesions is a source of bleeding in the chest wall, which is why hemostasis in these cases becomes even more important. Chest wall bleeding can hinder visualization of the surgical field, as well as causing considerable blood loss during surgery, or even lead to reoperation for the treatment of a retained hemothorax.

Dissection of the pulmonary hilum can also be a critical step in the operation. Due to the intense inflammatory process, the lymph nodes become more friable, and in chronic cases, calcified and adhered to the pulmonary hilum structures. In addition, fibrosis of the surrounding tissues cover the pulmonary hilum, making it difficult to dissect the pulmonary vessels and bronchus, increasing the risk of more severe intraoperative complications.

We therefore believe that the robotic platform can provide technical advantages in these cases. The 3D image of the robotic system provides better visualization of the intrathoracic structures and the notion of depth, enabling the lysis of adhesions under direct vision, with greater precision, resulting in less blood loss. The greater range of the robotic tweezers’ movements is useful in lysis of adhesions in the narrow upper chest, a noble chest area that contains the subclavian vessels and the brachial plexus. In addition, it allows the dissection of the pulmonary hilum with greater quality and safety when compared with VATS.

In our series, we use the Si platform due to its greater availability in hospitals. We inserted four portals (two for the robotic arms, one for the camera, and one for the assistant) as described by Terra et al.^9^. Our portal positioning and docking are similar to those proposed by Yablonskii et al.^10^. The difference is the location of the assistant portal, which we position in the 10th intercostal space anteriorly, triangulating with the camera arm and the robotic arm of the anterior portion. Yablonskii et al. advocate that a portal be positioned more posteriorly for an upper lobectomy, as it would facilitate the passage of the stapler for ligature of the pulmonary vein^10^. In our experience this detail was not actually relevant, nor has caused any technical difficulties for the team during the procedures.

It is worth mentioning that when available, the Xi system was used. We consider advantageous the possibility of using four robotic arms with lower collision incidence, providing greater independence to the surgeon at the console and lower variability of the surgical technique.

One of the main technical factors limiting the adoption of robotics platforms in operations for infectious lung disease is the presence of wide and firm pleuropulmonary adhesions, which may hinder or even prevent the positioning of the portals, the robotic arms, and, consequently, the robot docking. However, there are alternative strategies for solving this problem.

Upon identification of wide pleuropulmonary adhesions, the first step is to perform pulmonary detachment, just ample enough to facilitate the robot docking. For this, the assistant surgeon can perform the lysis of adhesions through an auxiliary portal using thoracoscopic maneuvers. This technique is primarily used to release adhesions of the diaphragm portion, due to the limitation of the robotic arms movement in this region.

Yablonskii et al.^10^ described an interesting strategy for pulmonary release in cases where there are dense adhesions to the diaphragm, named “re-docking procedure”. This consists of changing the target of the robotic platform system, which becomes the diaphragmatic portion instead of the apex of the pleural cavity. For this, the platform would be repositioned, making an angle of 175-185º with the patient’s head. The camera and assistant portals remain in their original positions, and the robotic clamps on the left and right sides are swapped. The Russian group advocates that the lysis of adhesions above the diaphragm is facilitated this way. At the end of this step, the platform is reset to the original position^10^. 

As for adhesions at the apex of the pleural cavity, these are more easily addressed using the robotic platform, mainly due to magnification and 3D image, as well as the tweezers great motion range. However, an interesting technical suggestion is that they should not be released initially, since these adhesions in the narrow upper chest allow good lung retraction, facilitating dissection of the pulmonary hilum. In addition, adhesions in these cases are usually rich in neovascularization and dissection in the initial phase of the operation can lead to bleeding and make it difficult to see the structures to be dissected^10^.

Our group has standardized the following surgical steps to complete the resection. We start the procedure with the release of the pulmonary ligament, followed by the posterior approach to the hilum with opening of the entire mediastinal pleura and lymph node dissection. We consider this a critical step, as good posterior dissection facilitates the next steps, such as the stapling of the pulmonary fissure^9^. Another important point is that inflammatory and/or infectious diseases are associated with anatomical distortion. In these cases, the lymph nodes function as a good anatomical parameter and guide the dissection, so that it is performed safely and efficiently.

Yablonskii et al.^10^ advocate starting the operation by the anterior approach of the lung hilum, since this way is more convenient to differentiate vascular anatomical abnormalities.

In our series of seven cases, we had no intraoperative complications or conversion to thoracotomy. This is due to the small number of patients, but also due to standardization of the technique and the accumulated experience of the group, which started in 2015. Currently, the team has performed over 500 robotic procedures. In the largest published series, with 53 cases, the Russian group presented two (4%) cases of conversions to thoracotomy, one due to firm adhesions in the pulmonary hilum and one injury to the pulmonary artery during the dissection of the fissure^10^.

The indication for conversion to thoracotomy is extremely important and should not be delayed when faced with great technical difficulties. We advocate that the procedure should be converted in cases where the surgeon is not comfortable and is aware that the dissection being performed is not safe. Conversion is no demerit, and the patient safety must always come first.

Our average total surgical time was 212 minutes, ranging from 100 to 375. This variation can be explained by the heterogeneity of the cases, in which some were more complex due to the large number of adhesions and inflammation. When analyzing only the average console time, there is a decrease to 147 minutes, varying from 61 to 288. This is due to two factors, one being the need of performing the adhesion lysis and pulmonary release by thoracoscopy before docking the robot itself, which delayed the start of the console use. The other is the time spent for hemostasis at the end of the operation, an even more important step in cases of great pleural detachment.

Yablonskii, the surgeon with the largest experience in robotic pulmonary resections for tuberculosis (53 cases), has a mean total and console surgical time of 175 min. and 109 min., respectively^10^. It is interesting that at the beginning of his experience, in the first 31 cases, the average total and console surgery time were 204 and 137 min., respectively^8^. This surgical duration was similar to ours, which indicates the importance of the learning curve in robotic lung resections for inflammatory and infectious diseases.

In our series, we had good operative results, without major complications. Our only complication was one case of constipation in the postoperative period, resolved with intestinal lavage. Our median times of chest drainage and hospital stay were one and five days, respectively, which are very acceptable figures for this patient profile. No patient required surgical rapprochement or hospital readmittance in the first 90 days after discharge.

 In the literature, we found an average drainage time of 3 ± 1 days and an 11% incidence of major complications, such as prolonged air leak, pleural effusion requiring puncture, and exacerbation of chronic obstructive pulmonary disease^10^.

The robotic thoracic surgery is already a reality in Brazil. In a short period, it attracted the interest of many surgeons and showed quick spread, especially in the private network. However, costs related to the purchase and maintenance of the robot, in addition to the purchase of materials, such as tweezers and disposable instruments, limit the implementation of a robotic surgery program in the public network, where there is a greater volume of patients with infectious and inflammatory lung diseases. To our knowledge, only one public hospital has the robotic platform. However, this is the center of reference in oncology, which can justify our study with seven patients during the study period.

Robotic surgery is an interesting alternative in the surgical treatment of infectious lung diseases. The platform offers several advantages to the surgeon for the performance of safer and higher quality procedures. Despite the small sample, we demonstrated that robotic thoracic surgery in infectious and/or inflammatory diseases is feasible, safe, and with a low risk of complications and morbidity.

The limitations of this work are the retrospective character and the absence of a sample calculation, being a technical feasibility study and description of the experience of a Brazilian group with a method that is still little explored in the literature for the cases of infectious / inflammatory lung diseases.
